# APPLYING REFRAMING AGING GUIDELINES WHEN REVIEWING FOR NON-AGING JOURNALS

**DOI:** 10.1093/geroni/igac059.2739

**Published:** 2022-12-20

**Authors:** Zainab Suntai

**Affiliations:** Baylor University, Waco, Texas, United States

## Abstract

The Reframing Aging initiative began in 2012 with the aim of changing the way in which the public views aging, and one of the key tenets of the initiative centers around language that perpetuates negative views of aging. Despite widespread knowledge about the initiative, recent publications in high-tier journals point to a gap in adopting the Reframing Aging initiatives outside of aging journals. Terms that project a negative view of older adults are still used in manuscript titles and within abstracts and bodies of research publications in non-aging journals. As researchers who publish aging-related work, members of organizations such as the Gerontological Society of America are often solicited as reviewers for their expertise in the field of aging. While many researchers in aging may review for aging-related journals, such expertise is often needed in non-aging journals. As such, it is critical for aging researchers to continue to advance the Reframing Aging initiative when conducting reviews of manuscripts that do not adhere to the guidelines. This presentation will provide explicit examples of such publications and review specific steps that reviewers can take in addressing the Reframing Aging initiative in future reviews.

